# Supplementary material for: Acute Myeloid Leukemia: A Key Role of DGKα and DGKζ in Cell Viability
Source: Cells. 2025 Nov 1;14(21):1721. doi: 10.3390/cells14211721 (PMC12609579; doi:10.3390/cells14211721)
Supplement: Supplementary file 1 [file cells-14-01721-s001.zip › Supplementary Data S3. Uncropped western blot images.pdf]

WB DGKα

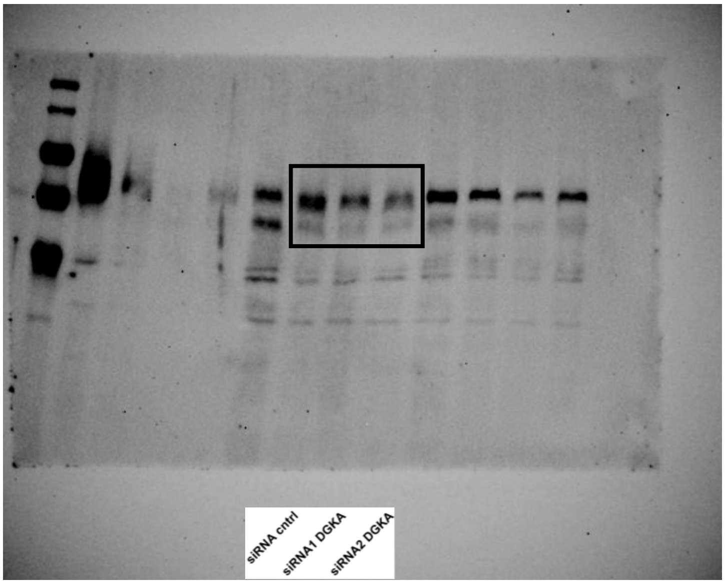

WB β-actin

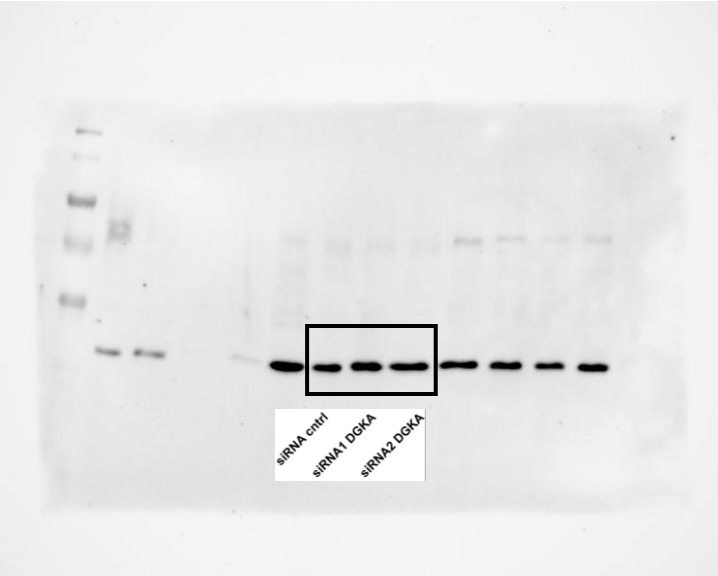

Figure 5: ***Uncropped gels***

WB DGKα

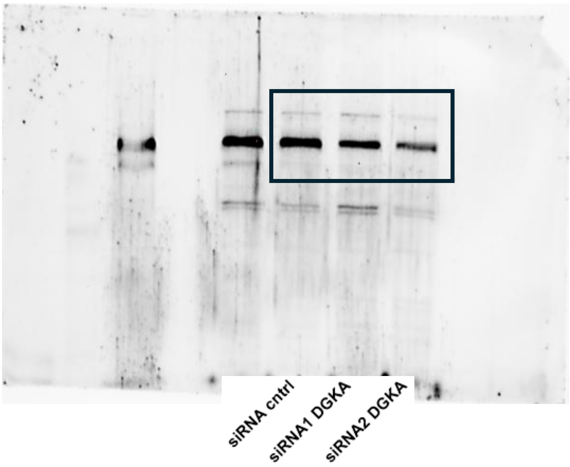

WB β-actin

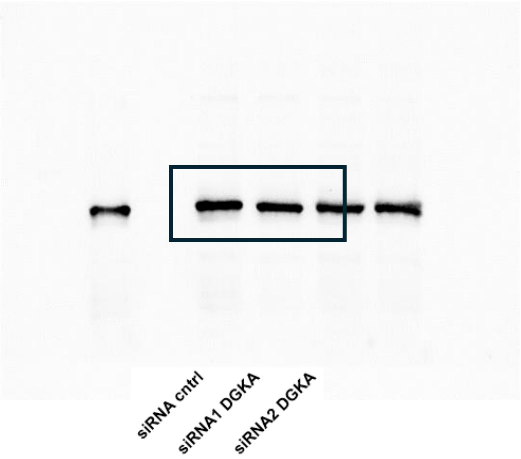

WB DGKζ

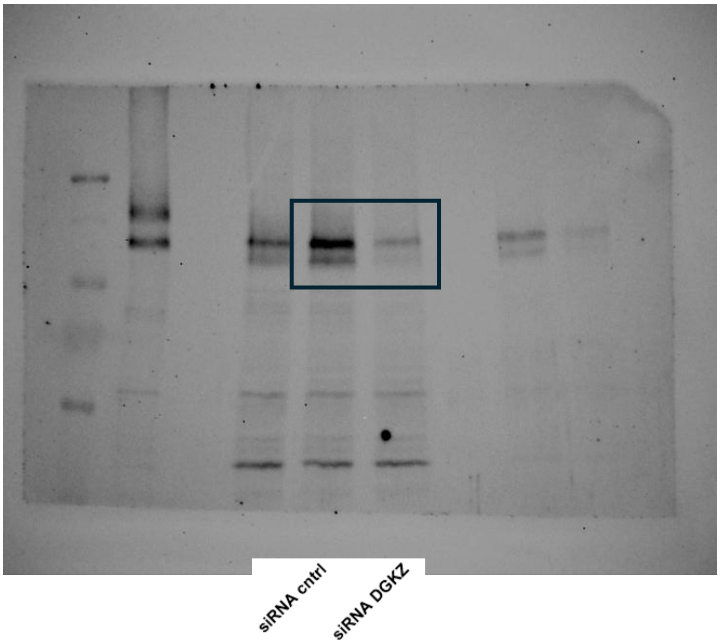

WB β-actin

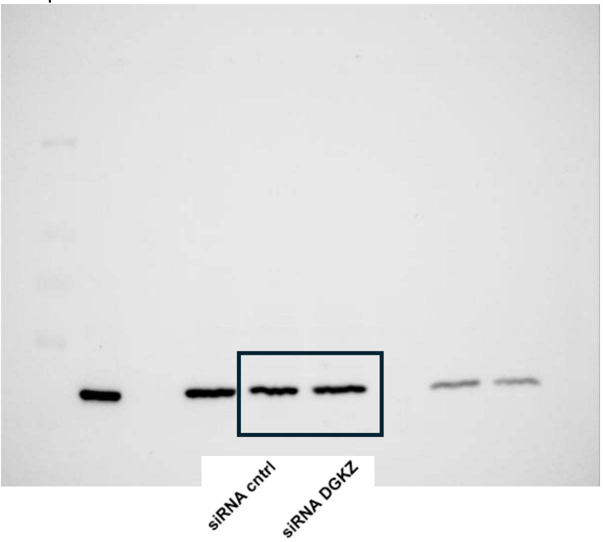

Figure 6: ***Uncropped gels***

WB DGKα

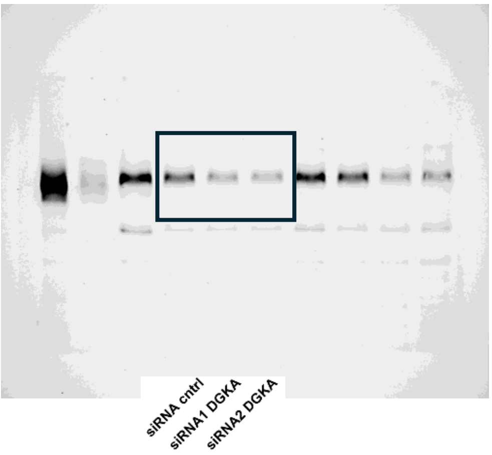

WB β-actin

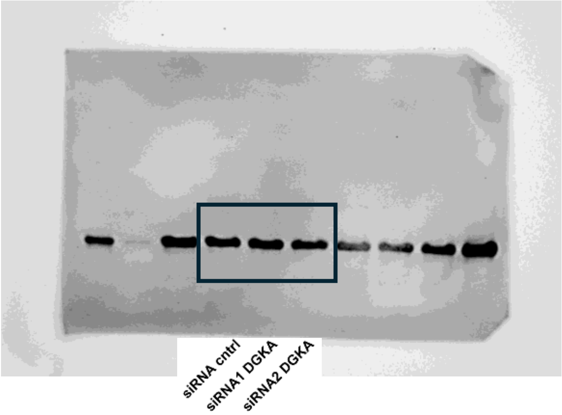

WB DGKζ

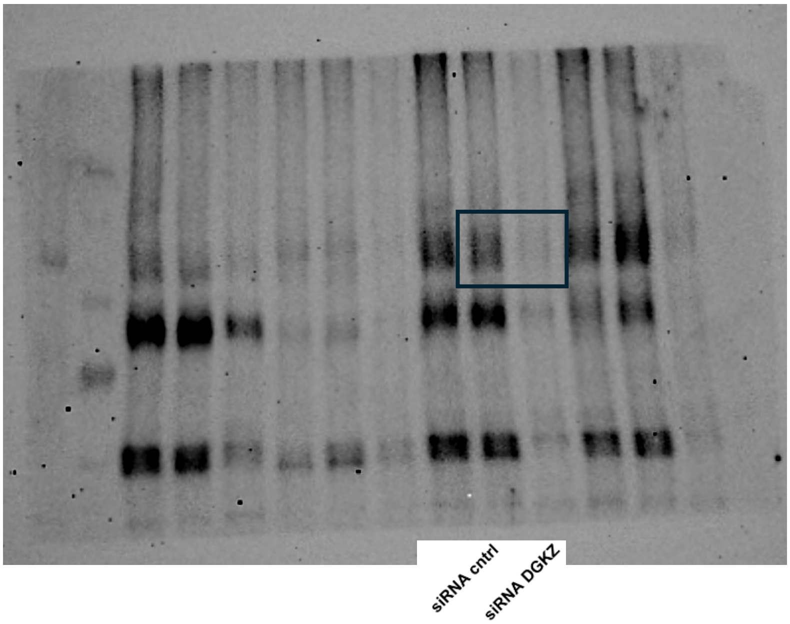

WB β-actin

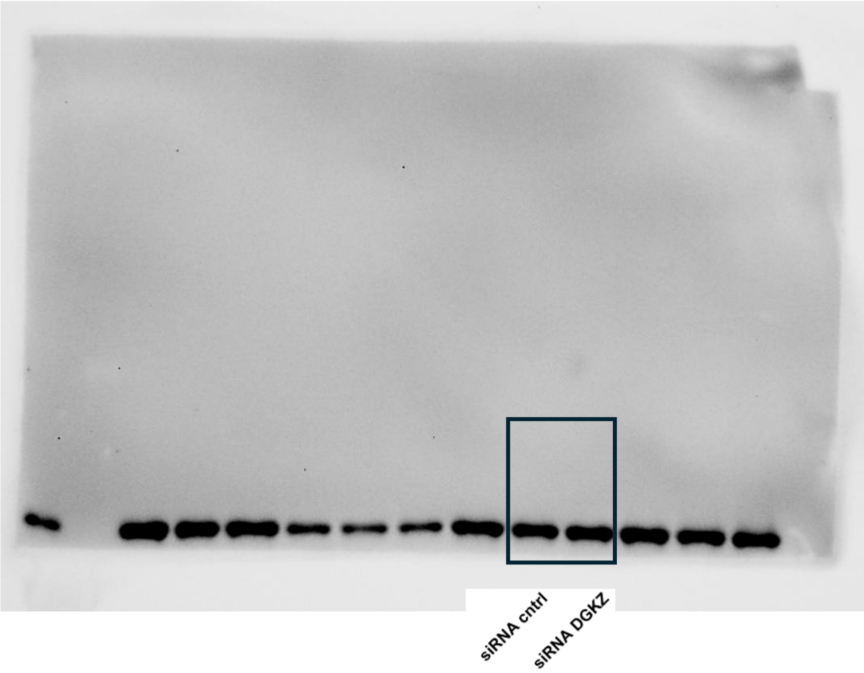

Figure 7: ***Uncropped gels***

WB DGKA

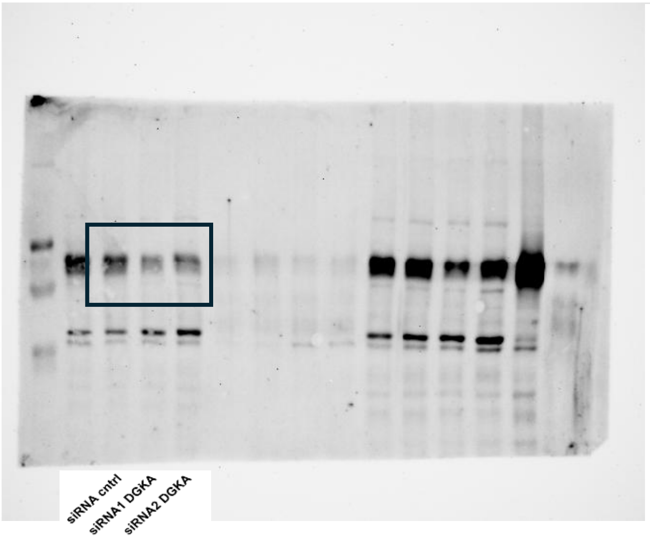

WB β-actin

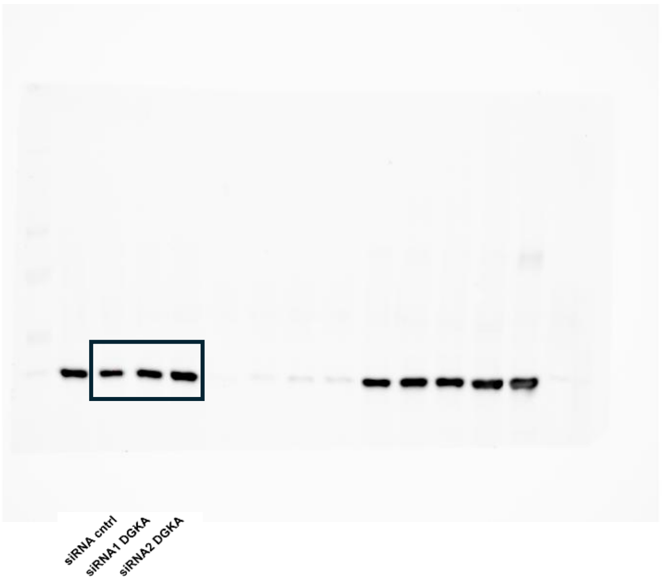

WB DGKZ

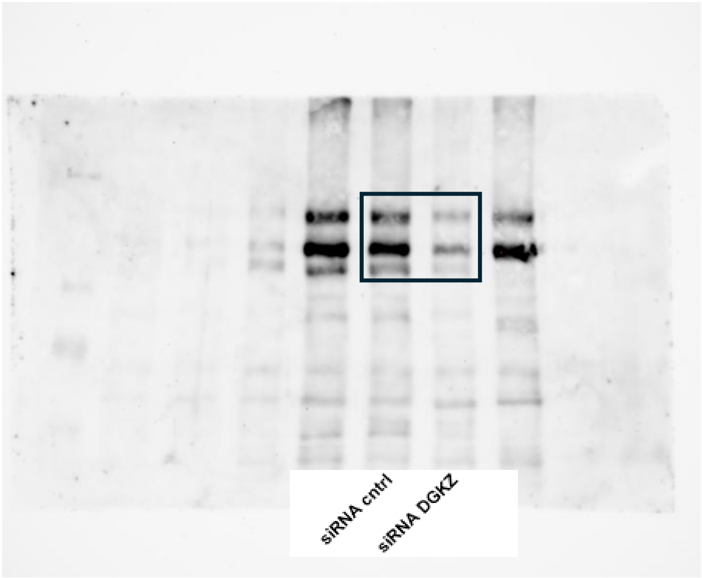

WB β-actin

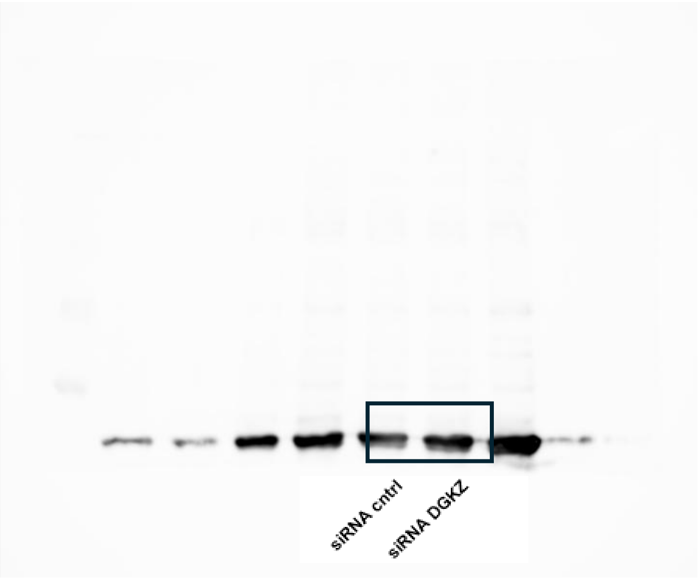

Figure 8: ***Uncropped gels***
