# Supplementary material for: Acute Myeloid Leukemia: A Key Role of DGKα and DGKζ in Cell Viability
Source: Cells. 2025 Nov 1;14(21):1721. doi: 10.3390/cells14211721 (PMC12609579; doi:10.3390/cells14211721)
Supplement: Supplementary file 1 [file cells-14-01721-s001.zip › Supplementary Figures S1-S8.pdf]

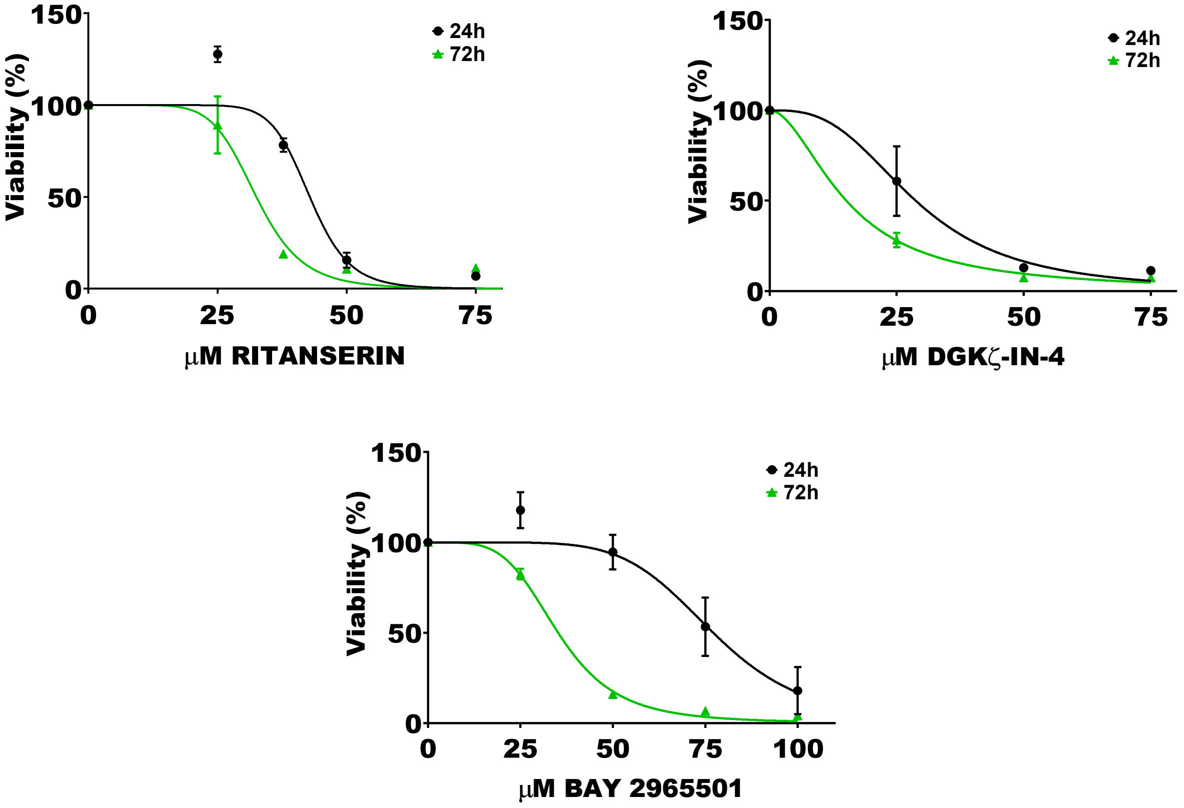


*Supplementary figureS1* : ***Effects of DGK inhibitors on primary cells viability.***

PBLs were treated with increasing concentration of ritanserin, DGKζ-IN-4 and BAY 2965501 for 24 or 72 hours followed by alamarBlue viability assay for additional 24 hours. Experiments were run in quadruplicates. Data are the mean ± SEM of 6 experiments interpolated as [inhibitor] vs % viability. Two-way ANOVA test, $p < 0.05$ *, $p < 0.01$ **, $p < 0.001$ *** and $p < 0.0001$ ****.

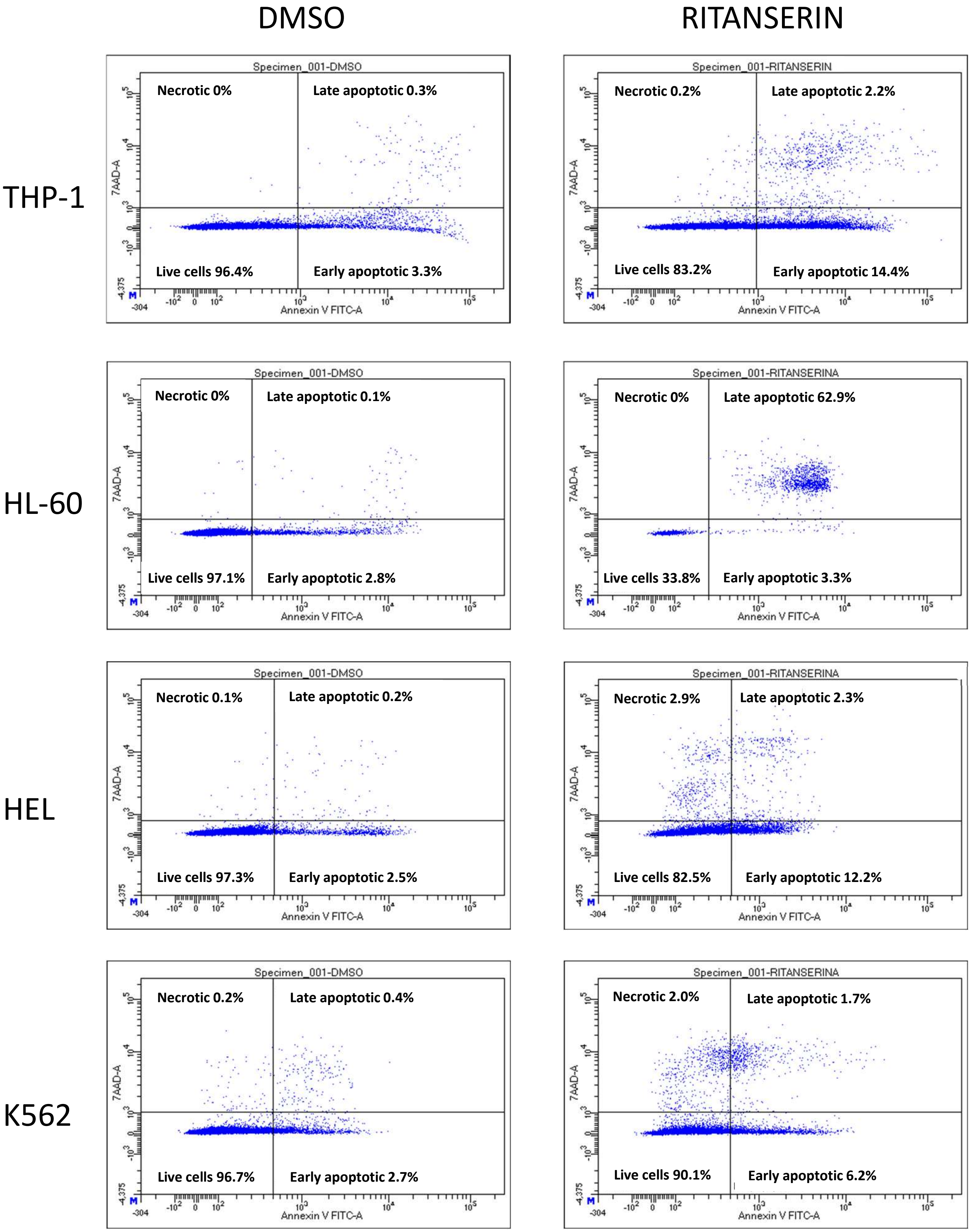


Supplementary figure S2: **Ritanserin effects on apoptosis/necrosis in AML cells**.
Cells were treated with ritanserin concentrations close to IC50. After 24 h, cells were stained with Annexin V-7AAD and analysed by flowcytometry to detect live (Annexin V-and 7AAD-), necrotic (Annexin-and 7AAD+), early apoptotic (Annexin V+ and 7AAD-) and late apoptotic (Annexin+ and 7AAD+) cell populations.

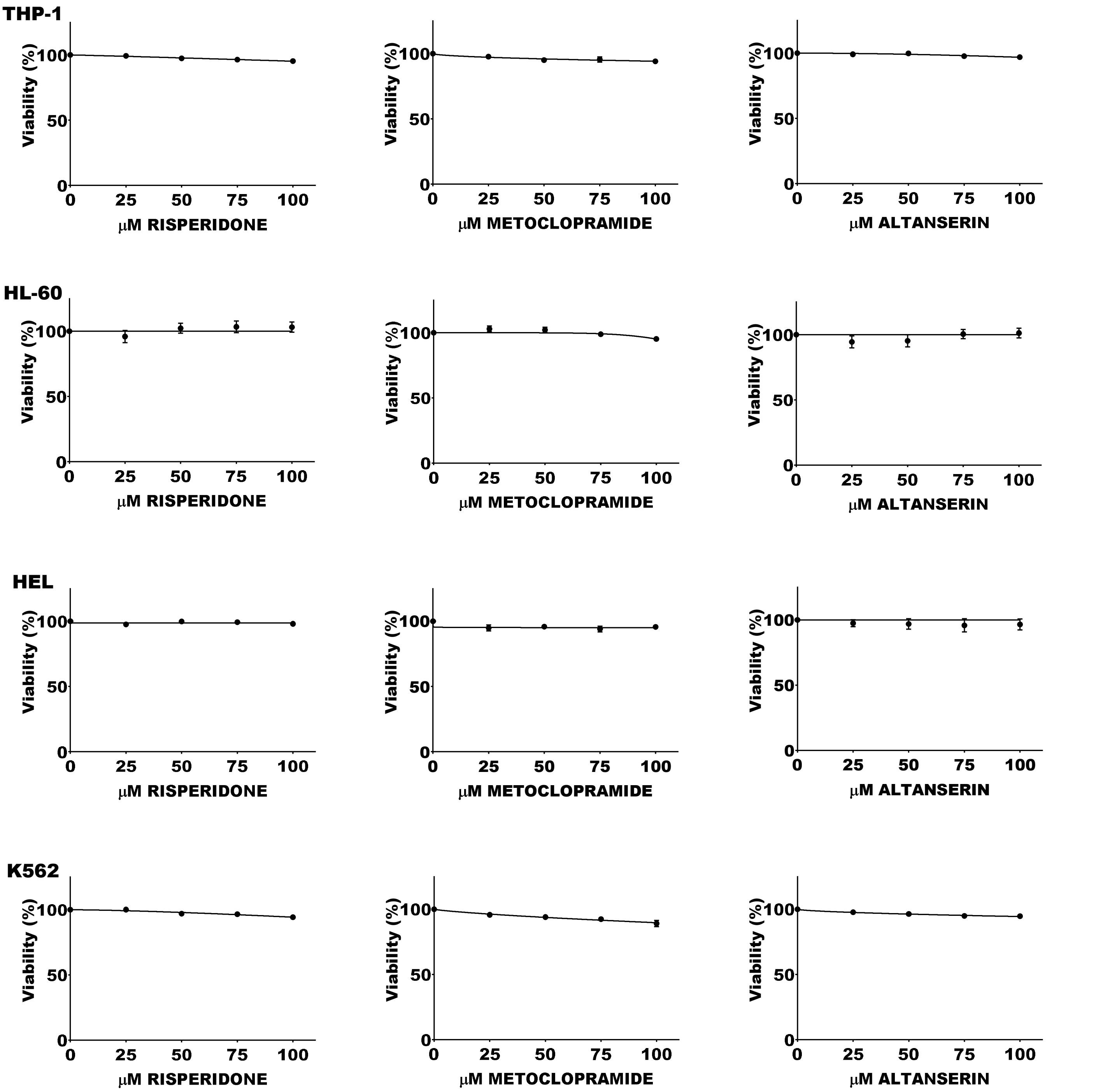


*Supplementary figure S3:* ***Effects of serotonin receptor inhibitors on AML cell lines viability.***
AML cells were treated with increasing concentration of the indicated inhibitor for 24 hours followed by alamarBlue viability assay for additional 24 hours. Experiments were run in quadruplicates. Data are the mean ± SEM of 4 or more experiments interpolated as [inhibitor] vs. % viability.

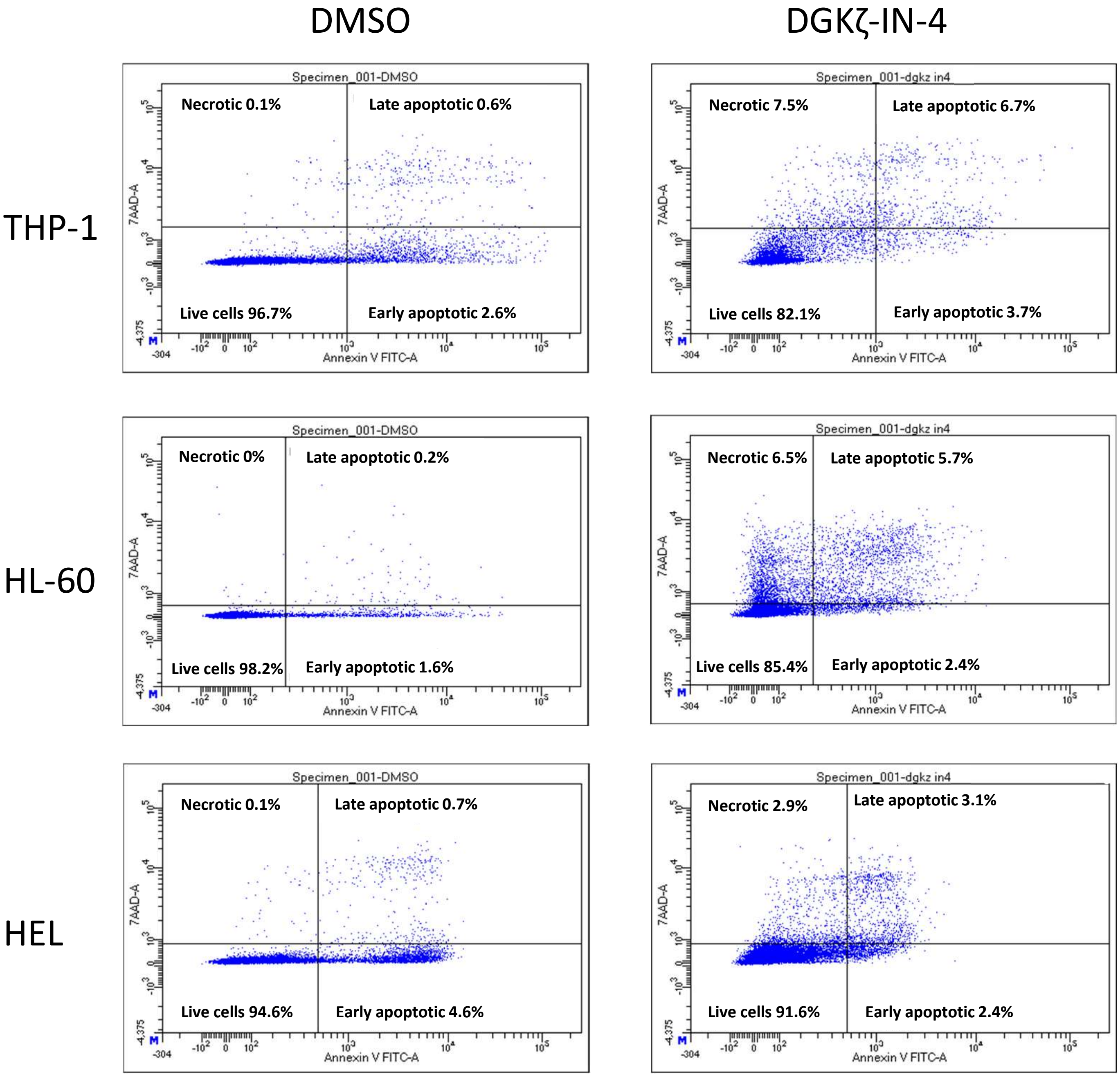


Supplementary figure S4: **DGKζ-IN-4 effects on apoptosis/necrosis in AML cells**.

Cells were treated with DGKζ-IN-4 concentrations close to IC50. After 24 h, cells were stained with Annexin V-7AAD and analysed by flowcytometry to detect live (Annexin V-and 7AAD-), necrotic (Annexin-and 7AAD+), early apoptotic (Annexin V+ and 7AAD-) and late apoptotic (Annexin+ and 7AAD+) cell populations.

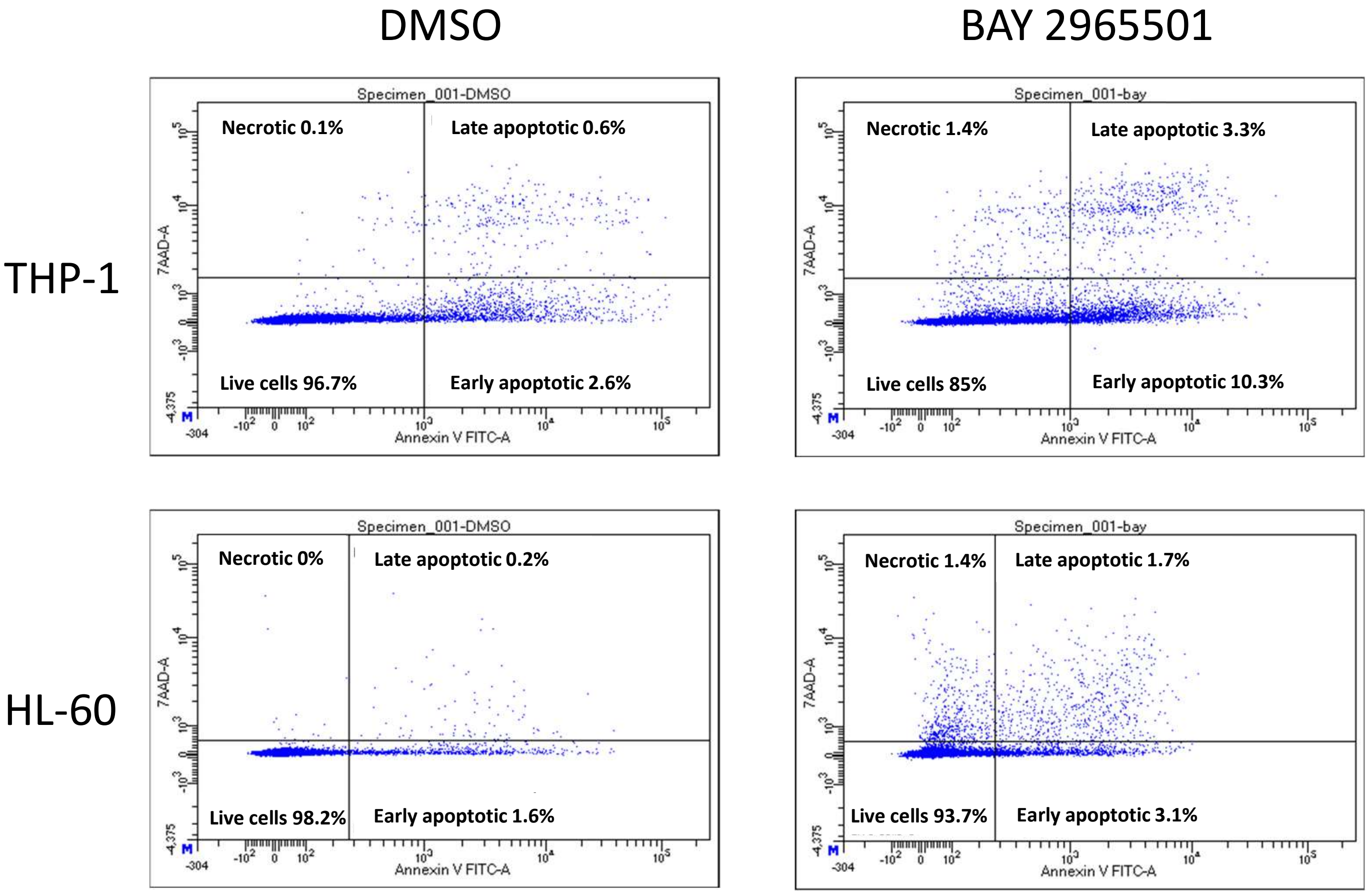


Supplementary figure S5: **BAY 2965501 effects on apoptosis/necrosis in AML cells**.
Cells were treated with BAY 2965501 concentrations close to IC50. After 24 h, cells were stained with Annexin V-7AAD and analysed by flowcytometry to detect live (Annexin V-and 7AAD-), necrotic (Annexin-and 7AAD+), early apoptotic (Annexin V+ and 7AAD-) and late apoptotic (Annexin+ and 7AAD+) cell populations.

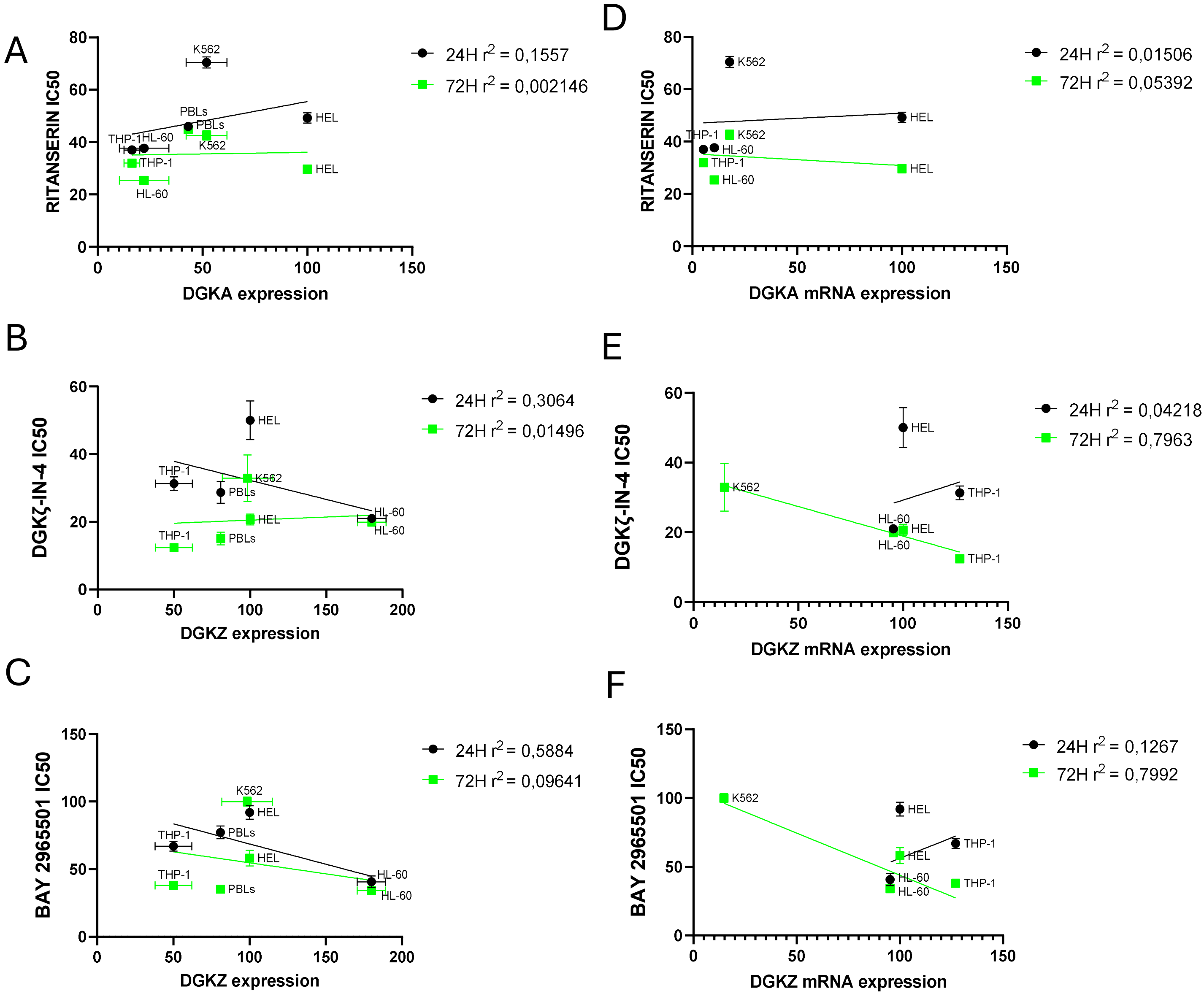


*Supplementary figure S6*: ***Correlation between sensitivity to isoform specific DGKs inhibitors and target expression.***

DGKs expression in PBLs and AML cell lines was measured by western blotting after normalization for protein concentration and rt-PCR after normalization for mRNA concentration. Expression is shown as percentage of HEL cells used as a reference.

A. Ritanserin IC50 vs DGKA protein;
B. BAY 2965501 IC50 vs DGKZ protein;
C. DGKζ-IN-4 IC50 vs DGKZ protein;
D. Ritanserin IC50 vs DGKA mRNA;
E. BAY 2965501 IC50 vs DGKZ mRNA;
F. DGKζ-IN-4 IC50 vs DGKZ mRNA.

Data were analysed using Graphpad prism linear regression. In B, C, E and F data for K562 cell line at 24h were excluded as IC50 is not defined.

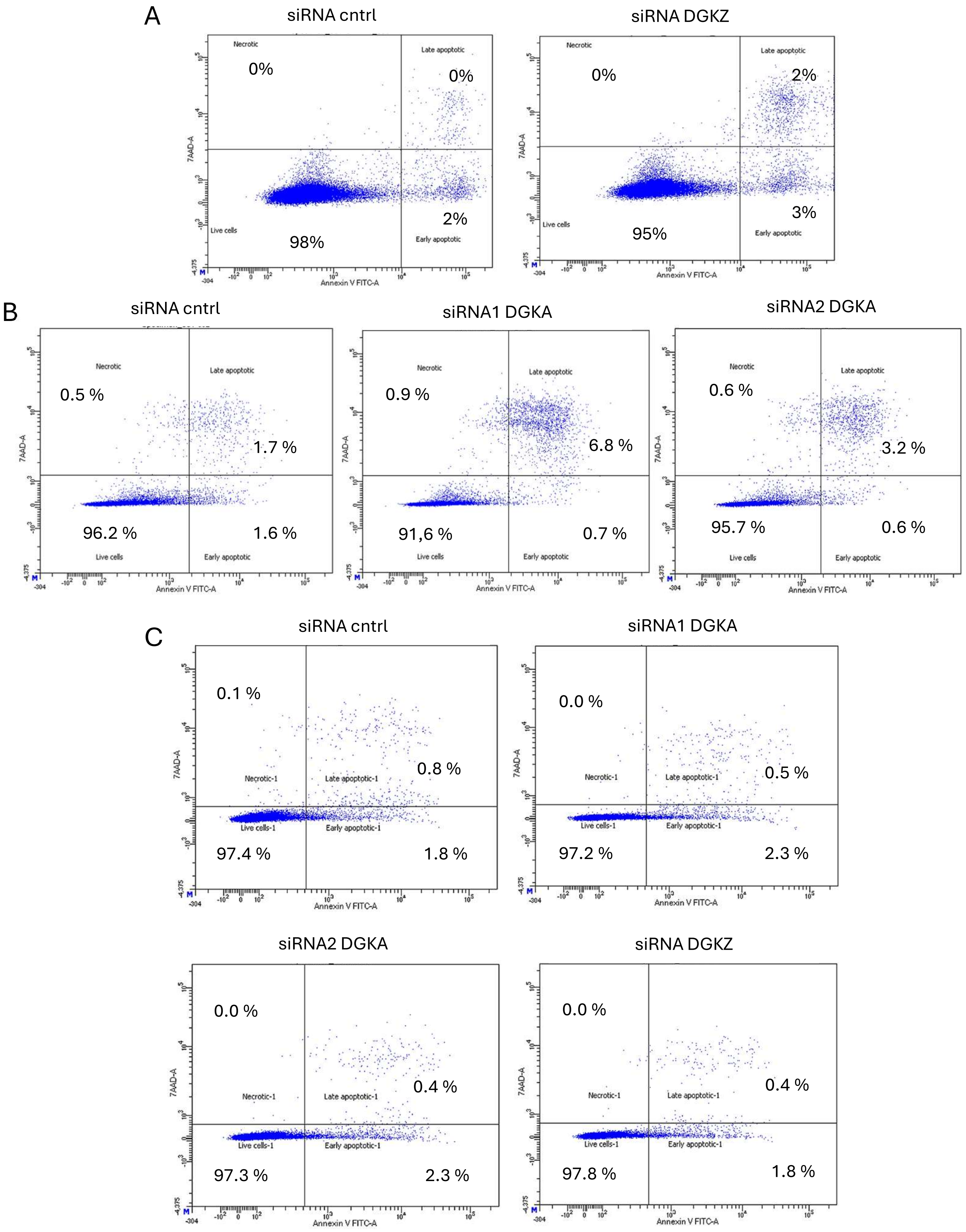


*Supplementary figure S7*: ***Apoptosis and necrosis after DGKA and DGKZ silencing in leukemia cell lines.*** Cells were silenced for either DGKA or DGKZ. After 72h, cells were stained with Annexin V-7AAD and analyzed by flow cytometry to detect live (Annexin V- and 7AAD-), necrotic (Annexin- and 7AAD+), early apoptotic (Annexin V+ and 7AAD-) and late apoptotic (Annexin+ and 7AAD+) cell populations.

A. Representative experiment on HL-60 cells;
B. Representative experiment on HEL cells;
C. Representative experiment on THP-1 cells.

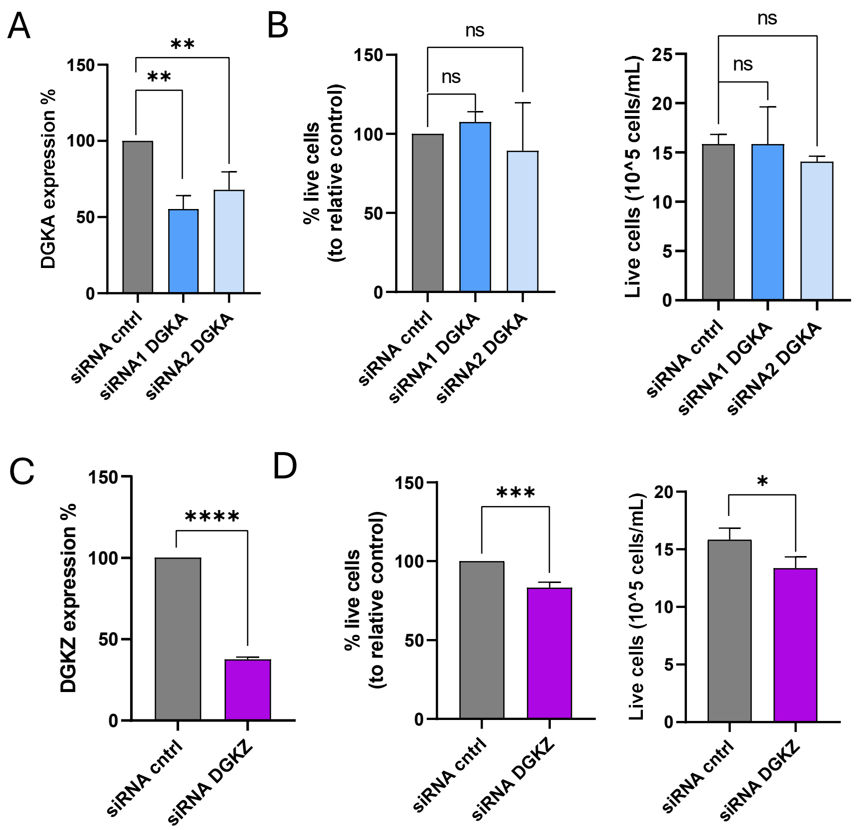


*Supplementary figure S8:* ***Role of DGKA and DGKZ on K562-related viability***

K562-related cells were transfected with the indicated siRNA and assessed after 72h.

A. Mean ± SEM of three independent experiments showing the silencing of DGKA on K562 cells. Data are presented as percentage of DGKA mRNA expression in comparison with siRNA cntrl transfected cells.
B. Trypan Blue assay. Mean ± SEM of three independent experiments shown as the percentage of live cells relative to siRNA cntrl (left). Right, a representative experiment showing the number of live cells starting from 15*10^5 cells/ml seeded.
C. Mean ± SEM of three independent experiments showing the silencing of DGKZ on K562 cells. Data are presented as percentage of DGKZ mRNA expression in comparison with siRNA cntrl transfected cells.
D. Trypan Blue assay. Mean ± SEM of three independent experiments shown as the percentage of live cells relative to siRNA cntrl (left). Right, a representative experiment showing the number of live cells starting from 15*10^5 cells/ml seeded.

The significance is assessed through two-way ANOVA or unpaired T test versus siRNA cntrl: $p < 0.05$ *, $p < 0.01$ **, $p < 0.001$ *** and $p < 0.0001$ ****.
