# Supplementary material for: Acute Myeloid Leukemia: A Key Role of DGKα and DGKζ in Cell Viability
Source: Cells. 2025 Nov 1;14(21):1721. doi: 10.3390/cells14211721 (PMC12609579; doi:10.3390/cells14211721)
Supplement: Supplementary file 1 [file cells-14-01721-s001.zip › Supplementary Tables S1 and S2.pdf]

REAGENT	CONCENTRATION	CAT. NUMBER	PROVIDER
RPMI-GlutaMAX 1640 Medium	-	61870-010	Thermo Fisher
Foetal bovine serum	-	A5256701	Thermo Fisher
Penicillin and Streptomycin	-	A5955	Sigma-Aldrich
rhIL-2	-	-	PeproTech
Trypan blue	0,4%	15250-061	Thermo Fisher
AlamarBlue	1,5 mg/mL in PBS	A50100	Invitrogen
Phosphate buffered saline	-	P4417	SIGMA
Triton X-100	-	04744977001	Roche Diagnostic
Dimethyl sulfoxide	1%	D2650	Sigma-Aldrich
Risperidone	-	R3030	Sigma-Aldrich
Altanserin	-	A8106	Sigma-Aldrich
Metoclopramide	-	M2218	TCI
Ritanserin	-	1955	Tocris
BAY 2965501	-	2732902-08-6	MedChemExpress
DGKζ-IN-4	-	HY-156574	MedChemExpress
Annexin V-FITC	-	BMS500FI-300	Invitrogen
7AAD	-	A9400-1MG	Sigma
DGK*α* Rabbit PolyAb	1 mg/mL	11547-1-AP	Proteintech
Rabbit Anti-DGKζ pAb	1 mg/mL	AB105195	Abcam
β-actin Monoclonal Antibody	1 mg/mL	MA1-140	Invitrogen
Rabbit Anti-Mouse IgG	1 mg/mL	A16160	Invitrogen
Goat Anti-Rabbit IgG	1 mg/mL	A16096	Thermo Fisher
PageRuler prestained protein ladder	-	26616	Thermo Fisher
High-Capacity cDNA Reverse Transcription Kit	-	4368814	Thermo Fisher
TaqMan™ Fast Advanced Master Mix	-	4444556	Thermo Fisher

Supplementary Table S1: materials used

NAME	TYPE	PRODUCER	CATALOG	ID	FORWARD	REVERSE
siRNA DGKZ	Custom siRNA	Ambion	AM16104	ACFARCW	GCCGCUUUCGGAAU AAGAUTT	AUCUUAUUCCGAAAGC GGCTG
siRNA1 DGKA	Custom siRNA	Invitrogen	10620310		AUAUUUAGCCAUC UCGCCAUCCUCG	CGAGGAUGGCGAGAU GGCUAAAUAU
siRNA2 DGKA	Custom siRNA	Invitrogen	10620310	494910C11	CGAGGAUGGCGAG AUGGCUAAAUA	AUAUUUAGCCAUCUCG CCAUCCUCG
siRNA cntrl	Silencer select	Invitrogen	465373			

Supplementary Table S2: siRNA used.
